# Acromegaly: a clinical perspective

**DOI:** 10.1186/s40842-020-00104-5

**Published:** 2020-08-20

**Authors:** Lima Lawrence, Kenda Alkwatli, James Bena, Richard Prayson, Varun Kshettry, Pablo Recinos, Betul Hatipoglu, Kevin M. Pantalone, Robert Weil, Amir H. Hamrahian, Laurence Kennedy, Divya Yogi-Morren

**Affiliations:** 1 Department of Endocrinology, Diabetes and Metabolism, Cleveland Clinic, 9500 Euclid Ave. F-20, Cleveland, OH 44195 USA; 2 Department of Quantitative Health Sciences, Cleveland Clinic, Cleveland, OH USA; 3 Department of Anatomic Pathology, Cleveland Clinic, Cleveland, OH USA; 4 Department of Brain Tumor and Neuro-Oncology Center, Cleveland Clinic, Cleveland, OH USA; 5 Lifespan Physician Group, Rhode Island Hospital, Providence, RI USA; 6 Department of Endocrinology, Diabetes and Metabolism, Johns Hopkins University, Baltimore, MD USA

**Keywords:** Acromegaly, IGF-1, Growth hormone, Pituitary adenoma, Sparsely granulated

## Abstract

**Background:**

To examine the clinical and hormonal profiles, comorbidities, treatment patterns, surgical pathology and clinical outcomes of patients diagnosed with acromegaly at the Cleveland Clinic over a 15-year period.

**Methods:**

A retrospective chart review of patients with acromegaly who underwent surgical resection between 2003 and 2018.

**Results:**

A total of 136 patients (62 men; mean age 48.1 years) with biochemical evidence of acromegaly were analyzed. Median insulin-like growth factor 1 (IGF-1) level at diagnosis was 769.0 ng/mL and most patients had a macroadenoma (82.2%). Immunoreactivity to growth hormone (GH) was noted in 124 adenomas, with co-staining in 89 adenomas. Complete visible tumor resection during initial surgery was achieved in 87 patients (64.0%). In this cohort, complete response to surgery alone was observed in 61 patients (70.1%), while 31 out of 65 patients (47.7%) who received additional post-surgical medications and/or radiation therapy achieved complete response. At most recent follow-up, 92 patients achieved eventual complete response by documented normalization of IGF-1 levels. Higher IGF-1 level at diagnosis (*P* = 0.024) and cavernous sinus invasion (*P* = 0.028) were predictors for failure to respond to surgery.

**Conclusion:**

In this study, the majority of tumors were macroadenoma, plurihormonal, and treated effectively with surgery alone or surgery with adjuvant medical or radiation therapy. More studies are needed to identify additional molecular biomarkers, tumor characteristics and imaging findings to individualize treatment and better predict treatment outcomes.

## Background

Acromegaly is a rare disease, characterized by excess growth hormone (GH) and insulin-like growth factor 1 (IGF-1) levels usually caused by a somatotroph adenoma of the pituitary gland. The elevated IGF-1 levels causes somatic growth and metabolic effects, with subsequently increased morbidity and mortality, particularly when GH and IGF-1 levels remain persistently elevated [1]. The presentation of acromegaly is insidious leading to diagnostic delays.

In patients with suspected acromegaly, the diagnosis is confirmed with biochemical tests including random serum IGF-1, and GH level following a glucose load. A pituitary magnetic resonance imaging (MRI) is performed to assess for evidence of an adenoma, followed by surgical resection as standard of care. About half of patients with macroadenoma do not achieve cure following resection and require repeat surgery, medications or radiation [2]. Medical management of acromegaly may involve use of somatostatin analogs (SSA), dopamine agonists (DA), growth hormone receptor antagonist pegvisomant (PEG) or combination therapy.

The aim of this study is to examine the clinical and hormonal profiles, comorbidities, complications, surgical pathology, treatment patterns, clinical outcomes, and mortality of patients diagnosed with acromegaly and surgically treated at the Cleveland Clinic over a 15-year period from 2003 to 2018.

## Methods

### Study design and patient selection

A retrospective chart review of all patients who were diagnosed with acromegaly in our institution over a 15-year period was undertaken. After approval from the Cleveland Clinic Institutional Review Board, we reviewed 1122 charts from a pituitary pathology database with surgically resected pituitary adenomas from 2003 to 2018. We selected patients from the pituitary pathology database who had a pituitary adenoma with evidence of GH staining on pathology, as well as patients who had clinical and biochemical evidence of acromegaly despite negative GH staining on pathology. Those who did not meet the above criteria were excluded. A total of 136 (12.1%) patients were identified for inclusion in the study.

We collected demographic information, clinical history including signs and symptoms at presentation, comorbidities, time since onset of symptoms and date of diagnosis. Additional data included biochemical lab results, tumor measurements, tumor characteristics on MRI, pathology information including pituitary hormone co-staining and granulation pattern, pre-operative medical treatment, completeness of initial tumor resection, medications initiated after surgery, and need for repeat surgery or irradiation. We also evaluated response to surgery, medication and radiation, along with patient-reported improvement in symptoms after treatment, and objective documentation of improvement in comorbidities. Complete initial tumor resection was defined as surgical removal of all visible tumor. Complete response to surgery was defined as radiographic absence of residual tumor and normalization of IGF-1 and GH levels (defined as random GH < 1.0 ng/mL) on last follow-up testing, while complete response to medications and irradiation therapy was defined as normalization of IGF-1 and GH levels on last follow-up testing regardless of residual tumor.

### Biochemical data

Laboratory data was mostly obtained from the Cleveland Clinic Main Laboratory, while some values were obtained from outside hospital records. Pre-operative data were obtained for serum GH (ng/mL), IGF-1 (ng/mL) and 2-h post oral glucose tolerance test (OGTT) GH (< 1.0 ng/mL). GH measurement was performed using quantitative chemiluminescence immunoassay (ARUP). Biochemical evidence of acromegaly was established with IGF-1 level above age-appropriate normal value, and nadir serum GH > 1 ng/mL post OGTT based on Endocrine Society and American Association of Clinical Endocrinologists (AACE) guidelines on acromegaly diagnosis. Although current GH assays have improved sensitivity, many assays do not have sufficient accuracy at GH levels < 1 ng/mL and there is poor reproducibility between laboratories and assays. As we reviewed laboratory data extending over 15 years from within and outside of our institution, we decided against using lower 2-h post-OGTT GH cutoffs in establishing the biochemical diagnosis of acromegaly.

### Imaging

Computed tomography (CT) or MRI reports were reviewed before and after surgical resection of pituitary adenoma. Based on the radiology report available in our electronic health record system or exported from outside institution, we obtained data on tumor diameter, tumor invasion and extension into cavernous sinus (CS) or optic chiasm (OC), and completeness of tumor resection post-surgery.

### Surgical procedures

All pituitary surgeries were performed by three dedicated skull-base neurosurgeons with or without involvement of an otolaryngologist. Surgical approaches included transsphenoidal approach with microscopy in earlier cases and endoscopic transnasal transphenoidal approach subsequently. In few select patients, repeat craniotomy (*n* = 3) was required. In all patients, the goal of surgery was gross total resection of all visible tumor, or maximal debulking in large and giant extended adenomas.

### Pathology evaluation

Surgical pathology was interpreted by neuropathologists at the Cleveland Clinic. Immunohistochemical staining with antibodies to pituitary hormones was routinely performed on pituitary adenomas. Antibodies evaluated included the following: anti-GH (1:2500 dilution; DAKO), anti-TSH (prediluted; Cell Marque, Rocklin, CA), anti-ACTH (1:2500 dilution; Thermo Scientific, Waltham, MA), anti-FSH (1:200 dilution; DAKO, Carpinteria, CA), anti-LH (1:320 dilution; DAKO) and anti-PRL (1:800 dilution; DAKO) with positive and negative control evaluated with each antibody stain in each case. Cytokeratin CAM5.2 staining (1:10 dilution; BD, San Jose, CA) was routinely used after 2015 to classify adenomas based on distribution pattern of keratin filaments into densely granulated (DG) or sparsely granulated (SG) adenomas. DG adenomas display a perinuclear or non-dot pattern with cytokeratin immunostaining under light microscopy; while SG adenomas show dot-like cytokeratin pattern with numerous CAM5.2 immunohistochemistry-positive fibrous bodies [3]. During data collection, based on pathology report, we classified GH staining as rare or diffuse, and if present, pituitary hormone co-staining evaluation for thyroid-stimulating hormone (TSH), prolactin (PRL), adrenocorticotropic hormone (ACTH) and follicle-stimulating hormone (FSH).

### Statistical analysis

For analysis of demographics, labs, tumor properties, evaluation of treatment and outcomes, categorical factors were summarized using frequencies and percentages, while continuous measures were evaluated for normality graphically and using the Shapiro-Wilk test. Normally distributed measures were summarized using means and standard deviations, while non-normal measures were summarized with medians and quartiles.

For univariable analyses, categorical factors were summarized using frequencies and percentages, and comparisons used Pearson’s chi-square or Fisher’s Exact tests, while continuous measures were evaluated for normality graphically and using the Shapiro-Wilk test. Normally distributed measures were summarized using means and standard deviations, and comparisons used ANOVA tests, while non-normal measures were summarized with medians, quartiles or ranges, and comparisons used Kruskal-Wallis tests. Odds ratios (OR) were calculated for non-zero responses.

A stepwise regression was performed for multivariable analyses for incomplete resection and persistent elevation in IGF-1 levels post-resection, given the low number of events for each outcome relative to the number of potential risk factors. Factors with a *P*-value less than 0.2 and at least 90% non-missing data were considered for the models. Variables were entered into the model if they had a *P*-value less than 0.10 and remained in the model if their P-value was under 0.05. After finalizing the stepwise methods, the models were refit with just the chosen variables to remove the impact of other variables on observations. OR and 95% confidence intervals were presented for each model. As a sensitivity analysis, penalized logistic regression using Firth’s methods was used to evaluate whether the models might be overfit.

A *P*-value of < 0.05 was predetermined as statistically significant. All models were fit and analyses were performed using SAS software (version 9.4; Cary, NC).

## Results

### Patients

Among the 1122 pituitary adenomas, 136 (12.1%) demonstrated biochemical evidence of acromegaly and 124 stained positive for GH on histology. Sixty-two men (45.6%) and 74 women (54.4%) were diagnosed at mean age of 48.1 ± 13.8 years. A majority of patients reported onset of symptoms at mean age 40.5 ± 13.8 years, with average diagnostic delay of 7.6 years. Available mortality data revealed 94.1% of patients were alive at the time of data collection based on our institution’s electronic health records.

Table 1 details the demographics and presenting symptoms. Musculoskeletal complaints (acral or facial enlargement, dental problems, osteoarthritis, carpal tunnel syndrome) were reported by 89.6% of patients, compressive symptoms (headache, visual changes or cranial nerve deficits) by 65.6% of patients, and hypogonadism (amenorrhea, sexual dysfunction, gynecomastia, galactorrhea) by 45.2% of patients. Miscellaneous concerns including hyperhidrosis, acne, hirsutism and dizziness were reported by 58.3% of patients, while 40.6% complained of psychological symptoms including mood changes and depression. Only a minority of patients (12.1%) were incidentally discovered to have a sellar mass on imaging as the presenting feature.

**Table 1:** Patient Demographics, Symptoms and Comorbidities at Presentation

Factor	Total (***N*** = 136)	
	n	n (%) or Mean ± SD
Sex	136	
• Male		62(45.6)
• Female		74(54.4)
Age at presenting symptoms	81	40.5 ± 14.2
Age at diagnosis	136	48.1 ± 13.8
Mortality status	136	
• Deceased		8(5.9)
• Alive		128(94.1)
Hypogonadism	126	57(45.2)
Compression symptoms	131	86(65.6)
Musculoskeletal symptoms	135	121(89.6)
Psychological symptoms	128	52(40.6)
^a^Miscellaneous symptoms	132	77(58.3)
Incidental CT/MRI finding	132	16(12.1)
Diabetes mellitus	133	59(44.4)
Hypertension	134	76(56.7)
Obstructive sleep apnea	122	58(47.5)
Coronary artery disease / congestive heart failure / cardiomyopathy	125	21(16.8)
Malignancy	132	17(12.9)
Genetic confirmation of MEN1 syndrome	136	1(0.74)

^a^Miscellaneous symptoms including hyperhidrosis, acne, hirsutism and dizziness

Patients presented with comorbidities including hypertension (56.7%), obstructive sleep apnea (47.5%) and diabetes mellitus (44.4%). Cardiac dysfunction including coronary artery disease, congestive heart failure or cardiomyopathy was observed in 16.8% of patients, while 12.9% had a history of, or were subsequently diagnosed with, malignancy (Table 1). Multiple endocrine neoplasia type-1 was confirmed with genetic testing in one patient.

Table 2 details the biochemical findings and tumor characteristics on presentation. Median (minimum to maximum range) IGF-1 level at diagnosis was 769.0 ng/mL (235 to 1845 ng/mL), with median baseline GH level of 8.2 ng/mL (0.17 to 808.12 ng/mL), and median nadir 2-h post-OGTT GH level of 4.4 ng/mL (0.2 to 330.0 ng/mL). Most patients were discovered to have a macroadenoma (82.2%), with median tumor dimensions of 16.0 (antero-posterior) × 16.0 (transverse) × 14.0 (craniocaudal) mm. CS invasion (44.7%) and OC compression (32.6%) were noted on MRI. GH staining was positive in 124 adenomas (91.2%). Out of 65 adenomas with reported staining pattern, 36 demonstrated diffuse staining for GH (55.4%), while 29 demonstrated rare GH staining pattern (44.6%). Pituitary hormone co-staining was observed in 89 adenomas (65.4%), including 57 adenomas staining for both TSH and prolactin (PRL), 12 staining for ACTH, and 4 staining for FSH. Cytokeratin CAM5.2 staining performed in 23 patients revealed 17 DG and 6 SG adenomas.

**Table 2:** Labs and Tumor Properties on Imaging and Histopathology

Factor	Total (***N*** = 136)	
	n	n (%) or Median [P25, P75]
Random GH level at Dx	125	8.2[3.2,34.0]
IGF-1 level at Dx	134	769.0[567.0,956.0]
2-h nadir post OGTT GH at Dx	44	4.4[1.6,16.9]
Most recent random GH level	128	0.61[0.31,1.8]
Most recent IGF-1 level	134	205.5[155.0,281.0]
Most recent 2-h nadir post OGTT GH	32	0.32[0.19,0.90]
Months between GH measures	116	21.5[7.9,65.3]
Months between IGF-1 measures	131	34.7[12.8,87.9]
Tumor size	135	
• Macroadenoma		111(82.2)
• Microadenoma		24(17.8)
Tumor length/antero-posterior	126	16.0[11.0,22.0]
Tumor width/transverse	116	16.0[10.5,20.0]
Tumor height/craniocaudal	105	14.0[10.0,20.0]
GH staining	136	124(91.2)
GH staining type	65	
• Rare		29(44.6)
• Diffuse		36(55.4)
Pituitary hormone co-staining	136	89(65.4)
• TSH	136	57(41.9)
• Prolactin	136	57(41.9)
• ACTH	136	12(8.8)
• FSH	136	4(2.9)
Granulation pattern	23	
• Densely		17(73.9)
• Sparsely		6(26.1)
Cavernous sinus invasion	132	59(44.7)
Optic chiasm compression	132	43(32.6)

### Treatment and outcomes

Eighty-four patients (62.7%) received pre-surgical medical treatment as follows: octreotide (80.9%) or lanreotide (7.1%) alone, followed by combination therapy with octreotide and cabergoline (4.8%), while 2 patients received PEG (Table 3). All 136 patients underwent pituitary tumor resection; four patients had initial craniotomy (2.9%), while 132 patients underwent transsphenoidal resection (97.1%). Complete visible tumor resection during initial surgery was achieved in 87 patients (64.0%). Subsequently, 19 patients required repeat surgery (of which three were open craniotomy), and 27 patients underwent irradiation.

**Table 3:** Treatment and Patient Outcomes

Factor	Total (***N*** = 136)	
	n	n (%)
Preoperative medical treatment	134	84(62.7)
• Octreotide	84	68(80.9)
• Lanreotide	84	6(7.1)
• Bromocriptine	84	1(1.2)
• Cabergoline	84	2(2.4)
• Pegvisomant	84	0
• Octreotide + Bromocriptine	84	1(1.2)
• Octreotide + Cabergoline	84	4(4.8)
• Octreotide + Pegvisomant	84	2(2.4)
Transsphenoidal surgery	136	135(99.3)
Craniotomy required	132	7(5.3)
Initial complete resection	135	87(64.4)
Repeat surgery	136	19(14.0)
Irradiation	135	27(20.0)
Medication initiated after surgery	136	65(47.8)
• Somatostatin analog (SSA)	65	32(49.2)
• Dopamine agonists (DA)	65	3(4.6)
• Pasireotide	65	0
• Pegvisomant (PEG)	65	1(1.5)
• Combination SSA + DA	65	8(12.3)
• Combination SSA + PEG	65	7(10.8)
• Combination DA + PEG	65	2(3.1)
• Sequential SSA + DA + PEG	65	11(16.9)
• Sequential SSA + DA + PEG + Pasireotide	65	1(1.5)
Response to surgery	87	61(70.1)
Response to medication and/or radiation:	65	31(47.7)
Hypopituitarism	133	22(16.5)
Improvement of symptoms after treatment	118	108(91.5)
Improvement of comorbidities after treatment	87	60(69.0)

Medical therapy post-surgery was initiated in 65 patients (47.8%), with 32 patients receiving SSA alone (49.2%), 3 patients receiving DA alone (4.6%), while 11 received combination therapy with SSA, DA and PEG (16.9%), and 1 patient received SSA, DA, PEG and pasireotide in step-wise escalation of therapy (Table 3). Complete visible tumor resection during initial surgery was achieved in 87 patients (64.0%). In this cohort, complete response to surgery alone was observed in 61 patients (70.1%), while 31 patients out of 65 (47.7%) who received subsequent post-surgical medications and/or radiation therapy achieved complete response. At most recent follow-up, 92 out of the total cohort of 136 patients (67.6%) achieved eventual complete response by documented normalization of IGF-1 levels. Overall, a significant majority of patients reported improvement in symptoms (91.5%) and had objective improvement in comorbidities (69.0%) after acromegaly treatment. The development of postsurgical hypopituitarism was noted in 22 out of 133 patients (16.5%). All patients who developed hypopituitarism had a history of irradiation, and 16 out of 22 patients (72.7%) with hypopituitarism had undergone repeat surgery.

### Univariable analyses with odds ratios

Table 4 details the outcomes of univariable analyses based on completeness of initial surgical tumor resection. Patients with complete initial resection of visible tumor had lower GH levels at diagnosis (*P* = 0.001) and smaller tumors in all dimensions (all *P* < 0.001). For each 1 ng/mL unit increase in GH, the odds of complete tumor resection decreased by 2% (OR (95% CI): 0.98 (0.97, 1.00), P = 0.001). Those with complete initial resection were also more likely to have GH staining (*P* = 0.022) and pituitary hormone co-staining (*P* = 0.026). They were less likely to have CS invasion and OC compression (both *P* < 0.001) or develop hypopituitarism (P < 0.001). The odds of a patient with hypopituitarism having had complete initial resection were 92% less than a patient without hypopituitarism (OR (95% CI): 0.08 (0.03, 0.27), P < 0.001).

**Table 4:** Labs and Tumor Properties of Patients with Complete Initial Resection

Factor	Incomplete (***N*** = 49)		Complete (***N*** = 86)			
	n	n (%) or Median [P25, P75]	n	n (%) or Median [P25, P75]	***p***-value	Odds Ratios(95% CI)
Random GH level at Dx	46	25.4[4.5,62.9]	78	6.3[2.6,21.1]	***0.001***^***b***^	0.98 (0.97, 1.00)
IGF-1 level at Dx	49	784.0[675.0,1014.0]	84	752.0[528.5944.0]	0.30^b^	1.00 (1.00, 1.00)
2-h nadir post OGTT GH at Dx	12	8.2[1.8,29.7]	31	4.3[1.7,12.8]	0.59^b^	1.00 (0.99, 1.01)
Most recent random GH level	45	1.4[0.32,2.2]	82	0.59[0.30,1.6]	0.11^b^	0.99 (0.96, 1.02)
Most recent IGF-1 level	49	217.0[153.0,377.0]	84	205.5[161.0,272.5]	0.50^b^	1.00 (1.00, 1.00)
Most recent 2-h nadir post OGTT GH	9	0.51[0.21,1.3]	23	0.30[0.16,0.80]	0.35^b^	0.74 (0.26, 2.1)
Tumor size	49		85		0.077^c^	
• Macroadenoma		44(89.8)		66(77.6)		reference
• Microadenoma		5(10.2)		19(22.4)		2.5 (0.88, 7.3)
Tumor length/antero-posterior	43	22.0[14.0,29.0]	82	14.5[10.0,18.0]	***< 0.001***^***b***^	0.92 (0.88, 0.96)
Tumor width/transverse	42	20.5[15.0,25.0]	74	14.5[10.0,18.0]	***< 0.001***^***b***^	0.89 (0.84, 0.94)
Tumor height/craniocaudal	39	20.0[15.0,25.0]	66	13.0[9.0,16.0]	***< 0.001***^***b***^	0.86 (0.80, 0.92)
GH staining	49	41(83.7)	86	82(95.3)	***0.022***^***c***^	4.0 (1.1, 14.1)
GH staining type	25		39		0.11^c^	
• Rare		14(56.0)		14(35.9)		reference
• Diffuse		11(44.0)		25(64.1)		2.3 (0.81, 6.3)
Pituitary hormone co-staining	49	26(53.1)	86	62(72.1)	***0.026***^***c***^	2.3 (1.10, 4.8)
• TSH	49	15(30.6)	86	41(47.7)	0.053^c^	2.1 (0.98, 4.3)
• Prolactin	49	16(32.7)	86	40(46.5)	0.12^c^	1.8 (0.86, 3.7)
• ACTH	49	2(4.1)	86	10(11.6)	0.14^c^	3.1 (0.65, 14.7)
• FSH	49	0(0.0)	86	4(4.7)	0.30^d^	NA
Granulation pattern	8		15		0.13^d^	
• Densely		4(50.0)		13(86.7)		reference
• Sparsely		4(50.0)		2(13.3)		0.15 (0.02, 1.2)
Cavernous sinus invasion	46	31(67.4)	85	28(32.9)	***< 0.001***^***c***^	0.24 (0.11, 0.51)
Optic chiasm compression	47	25(53.2)	84	17(20.2)	***< 0.001***^***c***^	0.22 (0.10, 0.49)

*p*-values: b = Kruskal-Wallis test, c = Pearson’s chi-square test, d = Fisher’s Exact test

Outcomes of univariable analyses based on response to surgery in those with complete initial resection were performed. Complete response to surgery was defined as normalization of IGF-1 levels without evidence of residual tumor at the time of last follow-up. Patients who responded to surgery had lower GH (*P* = 0.027) and lower IGF-1 levels (*P* = 0.001) at diagnosis, along with lower GH level (*P* = 0.031) and 2-h nadir post-OGTT GH level (*P* = 0.014) at most recent follow-up. Lastly, patients who responded to surgery were less likely to have CS invasion (*P* = 0.015). Outcomes of univariable analyses based on response to medication in those with complete initial resection were performed. Response to medication was defined as normalization of IGF-1 levels at last follow-up. Patients who responded to medication had lower levels of GH (*P* = 0.003) and IGF-1 (*P* = 0.046) at the time of their most recent follow-up.

Outcomes of univariable analyses of those who underwent repeat surgery were performed. Patients with repeat surgery were younger (*P* = 0.026), had higher GH levels at diagnosis (*P* = 0.011), larger tumors (anteroposterior *P* < 0.001, transverse *P* = 0.002, and craniocaudal P = 0.002), with CS invasion (*P* = 0.044) and OC compression (P = 0.011). Patients who underwent repeat surgery were less likely to have pituitary hormone co-staining with TSH (*P* = 0.047). Repeat surgery increased the risk of hypopituitarism (*P* < 0.001), with hypopituitarism present in 16 out of 19 patients (84.2%).

Outcomes of univariable analyses based on response to medication in those who had incomplete initial tumor resection were examined. Patients who responded to subsequent medical therapy were more likely to be female (*P* = 0.008), older at onset of symptoms and age at diagnosis (both *P* < 0.001), and less likely to report compressive symptoms on presentation (*P* = 0.022). These patients also had lower GH level (P < 0.001) at most recent follow-up. Outcomes of univariable analyses based on improvement in symptoms and comorbidities were performed. Patients who reported improvement in symptoms were more likely to be older at onset of symptoms (*P* = 0.036) and age at diagnosis (*P* = 0.023), and less likely to have hypopituitarism (*P* = 0.046). Patients who had objective improvement in comorbidities were more likely to be female and older at diagnosis (both *P* = 0.0.023). They had smaller tumor transverse dimension on presentation (*P* = 0.008) and less likely to have CS invasion (P = 0.008) and OC compression (*P* = 0.029). These patients also were more likely to have tumors stain positive for GH (*P* = 0.015) and co-stain with TSH (*P* = 0.036). Patients with improvement in comorbidities were less likely to have hypopituitarism (*P* < 0.001).

Subgroup analyses in Table 5 reveals that patients with SG adenomas were more likely to require repeat surgery than those with DG adenomas (*P* = 0.040).

**Table 5:** Outcomes of Patients with Densely and Sparsely Granulated Adenomas

Factor	Densely (***N*** = 17)		Sparsely (***N*** = 6)			
	n	n (%)	n	n (%)	***p***-value	Odds Ratios (95% CI)
Initial complete resection	17	13(76.5)	6	2(33.3)	0.13^d^	0.15 (0.02, 1.2)
Repeat surgery	17	1(5.9)	6	3(50.0)	***0.040***^***d***^	16.0 (1.2, 210.6)
Response to surgery	13	9(69.2)	2	0(0.0)	0.14^d^	NA
Response to medication and/or radiation:	5	2(40.0)	5	1(20.0)	0.99^d^	0.38 (0.02, 6.3)
Improvement of symptoms after treatment	16	16(100.0)	4	4(100.0)	N/A	N/A
Improvement of comorbidities after treatment	10	10(100.0)	3	3(100.0)	N/A	N/A

*p*-values: d = Fisher’s Exact test

### Multivariable analyses

Multivariable models for incomplete surgical resection and failure to respond to surgery among those with complete resection were analyzed. Predictors for failing to achieve complete initial tumor resection are CS invasion (*P* = 0.048) and OC compression (*P* = 0.023). Patients with CS invasion have 2.4 times higher odds of failure to achieve complete resection compared to patients without CS invasion, after adjustment for other terms in this model. Additionally, subsequent development of hypopituitarism is statistically correlated (*P* < 0.001) with incomplete initial tumor resection. Predictors of failure to respond to surgery after complete initial visible tumor resection are higher IGF-1 level at diagnosis (*P* = 0.024) and CS invasion (*P* = 0.028). Significantly, for each 10 ng/mL unit increase in IGF-1 at diagnosis, the odds of failure to respond to surgery increased by 2%, after adjusting for other factors. The development of hypopituitarism (*P* = 0.047) is correlated with failure to respond to surgery.

## Discussion

Our study demonstrates that patient demographics, initial biochemical findings, tumor characteristics and surgical pathology determine clinical outcomes in patients with acromegaly. The most prevalent presenting complaints and comorbidities in our study are consistent with current literature [4]. The overall low mortality rate (5.9%) mirrors the decline in acromegaly mortality rate over the past 30 years [5]. We observed a diagnostic delay of approximately 7 years, similar to findings reported in international acromegaly registries [6–8] and longitudinal studies [9]. Macroadenomas predominated in our cohort (82.2%) with associated CS invasion or OC compression, consistent with existing literature [4, 5, 9, 10].

The Endocrine Society and AACE guidelines on acromegaly management recommend pretreatment in a subset of acromegaly patients to improve postsurgical outcomes – for example, those with severe pharyngeal thickness, sleep apnea or high-output heart failure [11, 12]. In our study, a majority of patients received pre-operative medical therapy (62.7%) prior to surgical resection. Patients in our study who had complete visible tumor resection during initial surgery had lower levels of GH at diagnosis, smaller tumors, lacked CS invasion or OC compression, and were less likely to develop hypopituitarism. Factors associated with increased likelihood of surgical failure in our study is similar to findings in a large Korean study which noted that surgical failure was associated with younger age, larger tumor size and CS invasion [13]. European registries note a prevalence of hypopituitarism ranging from 26 to 34% in patients with acromegaly, which although higher than that in our study (16.5%), is an improvement from the past due to advances in surgical techniques, newer medications, and decreased use of radiotherapy [14–17]. A systematic review examining predictors of quality of life in acromegaly patients reported that hypopituitarism was not significantly correlated with quality of life [18]. However, our study found that patients with self-reported improvement in symptoms were significantly less likely to have hypopituitarism.

Cuevas-Ramos et al. proposed a structural and functional acromegaly classification partially based on granulation pattern: DG adenomas more commonly seen in older patients with longest follow-up and favorable outcomes and SG adenomas characterized by aggressive macroadenomas with adverse therapeutic outcomes despite multistep therapy [19]. In our study, we found that patients who required repeat surgery were significantly more likely to have SG adenomas (*P* = 0.040). Multiple studies have endorsed that patients with SG adenomas are younger, more likely to be female, with higher baseline IGF-1 and GH levels, have significantly larger tumors with aggressive features, and less likely to achieve biochemical normalization in response to surgery or SSA therapy, but exhibit therapeutic response to GH receptor antagonist therapy with PEG [20–26]. Therefore, GH adenoma granulation pattern may help guide acromegaly therapy. However, the current guidelines on the medical management of acromegaly continue to recommend first-generation long-acting SSA as first-line therapy in those with persistent disease after surgery, cabergoline as adjunct therapy, or transition to pasireotide or PEG based on glycemic status [11, 12, 27]. Therefore, updated guidelines that take into account the genetic, biochemical and histopathological variations in acromegaly, along with patient characteristics and imaging features that determine responsiveness to treatment are necessary. A recently published systematic review and consensus treatment guideline takes into account three factors in formulating a personalized approach to medical therapy of acromegaly: granulation pattern, somatostatin receptor 2 (SSTR2) staining and T2 intensity on MRI [28]. Based on their proposed decision tree, patients with DG tumors with high SSTR2 expressivity and T2 hypo-intense or iso-intense tumors on MRI would benefit from SSA therapy [28]. On the other hand, patients with SG tumors with low SSTR2 expressivity and hyper-intense T2-weighted MRI signal would benefit from DA therapy when there is only modest elevation of IGF-1, or PEG in the setting of significantly elevated IGF-1 levels, uncontrolled diabetes, and when tumor location and size is not a concern [28]. An accompanying commentary notes that this individualized approach to acromegaly is an improvement over the current “trial-and-assess” and “trial-and-failure” methods, avoiding prolonged futile and expensive treatments, and allowing earlier initiation of newer, more effective medications [29]. This personalized approach in acromegaly management is particularly relevant to those whose disease remains challenging to treat: patients with delayed diagnosis, ineligible for surgery, with persistent or recurrent disease after surgery [30].

Given the retrospective nature of this research, our analyses were primarily exploratory and carry with them potential limitations. From a statistical standpoint, the number of tests performed and use of stepwise modeling procedures both can inflate type I error rates and lead to spurious findings. However, we chose to include all analyses with raw, rather than adjusted, *p*-values so that the reader can use a preferred threshold for significance. Given these concerns, we recognize that these findings should be confirmed in other cohorts. Our report includes patients selected from a pituitary pathology database, which may be an underrepresentation of the total population of acromegaly patients treated at our institution who may have had surgical resection elsewhere, or were poor surgical candidates and were therefore managed medically or with irradiation therapy only. Although our study collected multiple variables, we were unable to quantify all factors which could have impacted choice of therapy and results. We also lack an objective method to assess patient compliance with therapy beyond physician documentation. Furthermore, we have inadequate numbers of DG and SG adenomas necessary to power statistically significant conclusions. However, strengths of our study include presentation of data from a robust number of patients with acromegaly treated in a tertiary care center for 15 years. Our enterprise-wide electronic health records provided a substantial amount of clinical data for us to collect and report.

## Conclusion

In this study, we report our experience with acromegaly in a large tertiary care center. Complete response to surgery alone was observed in 61 patients (70.1%), while 31 out of 65 patients (47.7%) achieved complete response with subsequent post-surgical medications and irradiation therapy. Predictors of failure to respond to surgery after complete initial resection were higher IGF-1 level at diagnosis and CS invasion. Patients with SG adenomas were more likely to require repeat surgery (*P* = 0.040); confirming the need for consideration of granulation pattern in individualizing the treatment of acromegaly.

## Data Availability

All data generated or analyzed during this study are included in this published article.

## Abbreviations

ACTH: Adrenocorticotropic hormone
CS: Cavernous sinus
DA: Dopamine agonist
DG: Densely granulated
FSH: Follicle-stimulating hormone
GH: Growth hormone
IGF-1: Insulin-like growth factor 1
LH: Luteinizing hormone
MRI: Magnetic resonance imaging
OC: Optic chiasm
OGTT: Oral glucose tolerance test
OR: Odds ratio
PEG: Pegvisomant
PRL: Prolactin
SG: Sparsely granulated
SSA: Somatostatin analog
SSTR2: Somatostatin receptor 2
TSH: Thyroid-stimulating hormone
